# Cystine/Glutamate Antiporter and Aripiprazole Compensate NMDA Antagonist-Induced Dysfunction of Thalamocortical L-Glutamatergic Transmission

**DOI:** 10.3390/ijms19113645

**Published:** 2018-11-19

**Authors:** Kouji Fukuyama, Toshiki Hasegawa, Motohiro Okada

**Affiliations:** 1 Department of Neuropsychiatry, Division of Neuroscience, Graduate School of Medicine, Mie University, Tsu 514-8507, Japan; mk_psy_isui@hotmail.com; 2 Department of Psychiatry, Mie University Hospital, Mie University, Tsu 514-8507, Japan; t-hasegawa@clin.medic.mie-u.ac.jp

**Keywords:** aripiprazole, schizophrenia, cystine/glutamate antiporter, metabotropic glutamate receptor, mediodorsal thalamic nucleus

## Abstract

To explore pathophysiology of schizophrenia, this study analyzed the regulation mechanisms that are associated with cystine/glutamate antiporter (Sxc), group-II (II-mGluR), and group-III (III-mGluR) metabotropic glutamate-receptors in thalamo-cortical glutamatergic transmission of MK801-induced model using dual-probe microdialysis. L-glutamate release in medial pre-frontal cortex (mPFC) was increased by systemic- and local mediodorsal thalamic nucleus (MDTN) administrations of MK801, but was unaffected by local administration into mPFC. Perfusion into mPFC of activators of Sxc, II-mGluR, and III-mGluR, and into the MDTN of activators of Sxc, II-mGluR, and GABA_A_ receptor inhibited MK801-evoked L-glutamate release in mPFC. Perfusion of aripiprazole (APZ) into MDTN and mPFC also inhibited systemic MK801-evoked L-glutamate release in mPFC. Inhibition of II-mGluR in mPFC and MDTN blocked inhibitory effects of Sxc-activator and APZ on MK801-evoked L-glutamate release; however, their inhibitory effects were blocked by the inhibition of III-mGluR in mPFC but not in MDTN. These results indicate that reduced activation of the glutamate/NMDA receptor (NMDAR) in MDTN enhanced L-glutamate release in mPFC possibly through GABAergic disinhibition in MDTN. Furthermore, MDTN-mPFC glutamatergic transmission receives inhibitory regulation of Sxc/II-mGluR/III-mGluR functional complex in mPFC and Sxc/II-mGluR complex in MDTN. Established antipsychotic, APZ inhibits MK801-evoked L-glutamate release through the activation of Sxc/mGluRs functional complexes in both MDTN and mPFC.

## 1. Introduction

Dysfunction of both dopaminergic and glutamatergic transmissions plays an important role in the pathophysiology of schizophrenia [1,2,3,4]. Dopaminergic dysfunction includes meso-limbic hyper-function and meso-cortical hypofunction of dopaminergic transmission [4]. Various atypical antipsychotics improve such paradoxical dopaminergic dysfunctions [5,6,7]. Indeed, an established antipsychotic drug, aripiprazole, 7-{4-[4-(2,3-dichlorophenyl)-1-piperazinyl] butoxy}-3,4-dihydro-2(1H)-quinolinone (APZ), has a high affinity for dopamine D2 and serotonin 5-HT1A receptors [8,9], and it can also act as a partial agonist for these same receptors [7,10,11]. Recently, we demonstrated that the interaction between the partial dopamine D2 and serotonin 5-HT1A receptors agonistic actions of APZ decreased and increased dopamine release in the meso-limbic (from the ventral tegmental area to nucleus accumbens) and meso-cortical (from the ventral tegmental area to prefrontal cortex) dopaminergic terminals, respectively [7].

The compelling link between glutamatergic transmission and schizophrenia is the ability of non-competitive glutamate/NMDA receptor (NMDAR) antagonists to induce schizophrenia-like positive and negative symptoms in healthy individuals [3,12,13], and to exacerbate psychosis in patients with schizophrenia [14]. Moreover, NMDAR antagonist-induced psychosis models exhibit certain features of schizophrenia, such as negative symptoms and cognitive deficits, more closely than the amphetamine/dopamine psychosis models [15]. Based on clinical and pre-clinical evidence, hypo-glutamatergic transmission via the inhibition of NMDAR seems sufficient to produce a schizophrenia-like state.

Previous pre-clinical studies demonstrated that systemic administration of NMDAR antagonists increased extracellular L-glutamate levels in the medial prefrontal cortex (mPFC), resulting in an increased firing rate of cortical neurons [16,17], and that these changes were necessary for the psychotomimetic effects of phencyclidine and ketamine [17,18,19]. Thus, the precise role of glutamatergic transmission is equivocal, since both an increase and decrease in glutamatergic signaling contribute to the symptoms of schizophrenia. Furthermore, the exact patho-mechanism of glutamatergic dysfunction remains to be elucidated. Contrary to systemic administration, however, local administration of a non-competitive NMDAR antagonist in the mPFC did not affect extracellular L-glutamate levels in that region [5,6,7,20,21,22]. The discrepancy between the effects of systemic and mPFC local administrations of NMDAR antagonists on the glutamatergic transmission in the mPFC suggests that the fundamental region of NMDAR antagonist-induced frontal hyper-glutamatergic transmission is probably outside the mPFC. In this regard, mPFC receives two major glutamatergic projections: one from other cortical regions and the other from the mediodorsal thalamic nucleus (MDTN) [5,6,23,24,25]. Therefore, neurotransmission between the MDTN and mPFC (thalamo-cortical glutamatergic pathway) could also play an important role in the pathophysiology of schizophrenia, mood disorders, and epilepsy [5,6,26,27,28].

Recent clinical studies have suggested that the activation of system xc- (Sxc: a cystine/glutamate antiporter) through the systemic administration of a cyst(e)ine prodrug, N-acetyl-L-cysteine (NAC) improved cognitive impairment in schizophrenia and ketamine-induced psychosis [29]. Pre-clinical studies indicated that NAC inhibited L-glutamate release in the mPFC induced by systemic administration of phencyclidine [19]; however, Sxc extra-synaptically releases L-glutamate via exchanges of extracellular L-cystine and intracellular L-glutamate across the cellular plasma membrane of astrocytes [30]. Thus, the inhibitory effects of NAC on behavior abnormalities and the hyper-activation of glutamatergic transmission in the mPFC induced by an NMDAR antagonist is probably mediated by the other transmission mechanism, i.e., metabotropic glutamate receptors (mGluRs), since NAC enhances the activity of group II metabotropic glutamate receptors (II-mGluR) [31,32]. Furthermore, Sxc positively interacts with group III metabotropic glutamate receptors (III-mGluR) in non-neuronal cells [33]. Therefore, if a functional complex between Sxc and mGluRs regulates thalamo-cortical glutamatergic transmission, the modulation of a Sxc/mGluR functional complex provides the strategies for the novel medications against cognitive impairments of schizophrenia.

Based on the above background, the present study was designed to further understand the pathophysiology of schizophrenia, with a special focus on thalamo-cortical glutamatergic hyper-function (hyper-glutamatergic hypothesis). For this purpose, the following three major experiments were conducted, using dual probe microdialysis with ultra-high-performance liquid chromatography (UHPLC). The present study particularly compared the effects of systemic and local administration (into the MDTN and mPFC) of a non-competitive NMDAR antagonist, 5-methyl-10,11-dihydro-5H-dibenzo[a,d]cyclohepten-5,10-imine (MK801) and any agents that are associated with Sxc and mGluRs on thalamo-cortical glutamatergic transmission, to avoid any influences from other neuronal pathways on this glutamatergic pathway. (1) To explore the candidate fundamental neuronal pathway of NMDAR antagonist-induced L-glutamate release in the mPFC, the effects of systemic and local administrations of MK801 into the MDTN and mPFC on L-glutamate release in the mPFC were studied. (2) To explore the regulation mechanisms of glutamatergic transmission in the thalamo-cortical pathway, the interaction between Sxc, mGluRs, and MK801 in the MDTN and mPFC on thalamo-cortical glutamatergic transmission were determined. (3) To explore whether the established antipsychotics affect thalamo-cortical glutamatergic transmission, the interaction between APZ and inhibitors of Sxc and mGluRs was studied, since APZ inhibits schizophrenia-like behavior and mPFC L-glutamate release induced by the systemic administration of NMDAR antagonists [34,35].

## 2. Results

The present study was composed of seven experimental designs according to the drug administration roots. Each figure represented the data obtained from a corresponding experimental design. Rats were randomly assigned to treatment in each experimental design. Where possible, we sought to randomize and blind the sample data. In particular, for the determination of the extracellular L-glutamate level, each sample was set on an auto sampler, according to a table of random numbers. Once eight consecutive baseline levels of L-glutamate were stable. When the coefficients of variation for each L-glutamate had reached less than 5% over 60 min (stabilization), dialysate was collected for 60 min as pretreatment periods of MK801, and for 180 min as post-MK801-administration periods.

### 2.1. Effects of Systemic Administrations of MK801 and NAC on Extracellular L-Glutamate in mPFC (Study 1)

**Figure 1:**
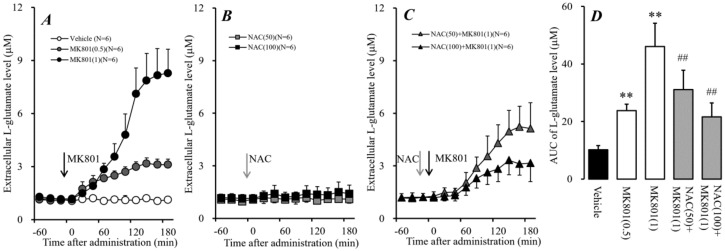
Effects of 5-methyl-10,11-dihydro-5H-dibenzo[a,d] cyclohepten-5,10-imine (MK801) and N-acetyl-L-cysteine (NAC) on extracellular L-glutamate in the medial pre-frontal cortex (mPFC). (**A**) indicates the effects of systemic administration of MK801 (0.5 and 1 mg/kg, i.p.) on L-glutamate release in the mPFC. (**B**) indicates the effects of systemic administration of NAC (50 and 100 mg/kg, i.p.) on L-glutamate release in the mPFC, compared with the vehicle from (**A**). (**C**) indicates the effect of NAC (50 and 100 mg/kg, ip) on systemic MK801-evoked L-glutamate release in the mPFC, compared with MK801 alone (1 mg/kg, i.p.) from (**A**). Black and gray arrows indicate the intraperitoneal injection of MK801 and NAC, respectively. Microdialysis was conducted to measure the L-glutamate release in the mPFC. In (**A**–**C**), ordinates: mean ± SD (*n* = 6) of extracellular L-glutamate level in the mPFC (μM), abscissa: time after administration of MK801 (0.5 and 1 mg/kg) or NAC (50 and 100 mg/kg) (min). (**D**) indicates the area under curve (AUC) value of extracellular L-glutamate level in the mPFC (μM) after drug injection from 0 to 180 min of (**A**–**C**). ** *p* < 0.01; relative to vehicle (black) and ## *p* < 0.01; relative to MK801 (1 mg/kg, i.p.) (open) using the linear mixed effect model (LMM) with Tukey’s post hoc test.

Systemic administration of NMDAR antagonists phencyclidine and ketamine increased extracellular L-glutamate levels in the mPFC [16,17], and recent clinical studies demonstrated that cysteine prodrug (Sxc activator) NAC improved cognitive impairments in ketamine-induced psychosis [29]. These previous demonstrations suggest the NAC probably antagonizes the elevation of L-glutamate release induced by an NMDAR antagonist. However, Sxc is widely expressed in astrocytes and releases L-glutamate [30]. Therefore, to determine the effects of dose-dependent systemic administrations of a non-competitive NMDAR receptor antagonist, MK801 (0.5 and 1 mg/kg), NAC (50 and 100 mg/kg), and interaction between MK801 (1 mg/kg) and NAC (50 and 100 mg/kg) on the extracellular L-glutamate level in the mPFC, MK801, and NAC were injected intraperitoneally during perfusion with a modified Ringer’s solution (MRS) into the mPFC (Figure 1).

Systemic administration of MK801 (0.5 and 1 mg/kg, i.p.) dose-dependently increased the extracellular L-glutamate level in the mPFC [F_MK801_(2,15) = 78.5 (*P* < 0.01), F_time_(9,135) = 155.0 (*P* < 0.01), F_MK801*time_(18,135) = 88.8 (*P* < 0.01)], whereas systemic administration of NAC (50 and 100 mg/kg, i.p.) did not affect the extracellular L-glutamate level in the mPFC (Figure 1A,B,D). Systemic administration of NAC alone did not affect the extracellular L-glutamate level in the mPFC; however, NAC (i.p.) inhibited the release of L-glutamate in the mPFC induced by systemic administration of 1 mg/kg MK801 (i.p) [F_NAC_(2,15) = 19.9 (*P* < 0.01), F_time_(9,135) = 170.2 (*P* < 0.01), F_NAC*time_(18,135) = 20.1 (*P* < 0.01)] (Figure 1C,D). Both demonstrations, MK801-induced mPFC L-glutamate release and the inhibition of this L-glutamate release by NAC, which improves NMDAR antagonist-induced cognitive impairment, suggest that the pathophysiology of cognitive impairments is induced by the hyper-activation of glutamatergic transmission in the mPFC.

### 2.2. Concentration-Dependent Effects of Perfusion with MK801 into mPFC and MDTN on L-Glutamate Release in mPFC (Study 2)

**Figure 2:**
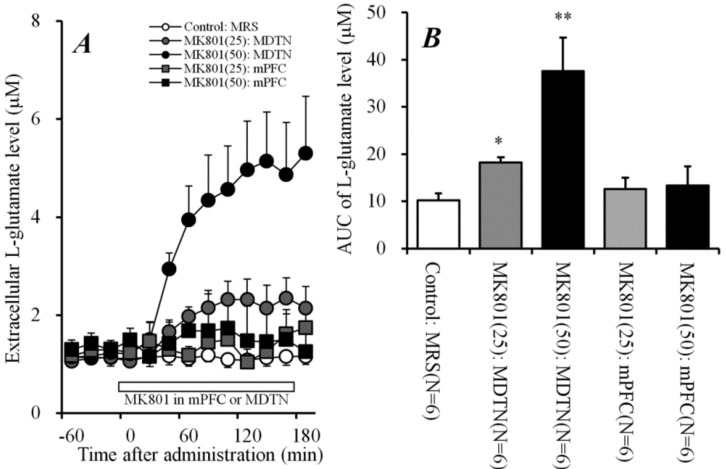
Concentration-dependent effects of local administration of MK801 into the mPFC and mediodorsal thalamic nucleus (MDTN) on L-glutamate release in the mPFC. (**A**) indicates the comparison of the concentration-dependent effect of perfusion with MK801 (25 and 50 μM) into the MDTN and mPFC on L-glutamate release in the mPFC. In (**A**), ordinate: mean ± SD (*n* = 6) of the extracellular L-glutamate level in the mPFC (μM), abscissa: time after administration of MK801 (min). Open bars: perfusion of MK801. (**B**) indicates mean ± SD (*n* = 6) of AUC value of the extracellular L-glutamate level in mPFC (μM) during perfusion with MK801 (25 and 50 μM) into the MDTN (gray columns) and into the mPFC (opened columns) from 0 to 180 min of (A). * *p* < 0.05, ** *p* < 0.01; relative to control (MRS alone) using LMM with Tukey’s post hoc test.

Study 1 demonstrated that systemic MK801 administration dose-dependently increased the extracellular L-glutamate level in the mPFC, whereas the inhibition of NMDAR in the mPFC did not affect extracellular L-glutamate levels in that region [5,6,7,20,21,22]. Therefore, to explore the fundamental brain regions (outside the mPFC) of L-glutamate release in the mPFC induced by systemic administration of MK801 (systemic MK801-evoked L-glutamate release), Study 2 determined the concentration-dependent effects of local administration of MK801 into the mPFC and MDTN on the extracellular L-glutamate level in the mPFC (Figure 2).

Perfusion with MK801 (25 and 50 μM) into the mPFC had no effect on the extracellular L-glutamate level in the mPFC, similar to previous studies [5,6,7,20]; however, perfusion of MK801 (25 and 50 μM) into the MDTN concentration-dependently increased the extracellular L-glutamate level in the mPFC [F_MK801_(2,15) = 62.7 (*P* < 0.01), F_time_(9,135) = 79.9 (*P* < 0.01), and F_MK801*time_(18,135) = 37.9 (*P* < 0.01)] (Figure 2A,B). Therefore, the MDTN is a candidate generator region of systemic MK801-evoked L-glutamate release in the mPFC.

### 2.3. Effects of Perfusion of Activators of Sxc, mGluRs, and GABA_A_ Receptor into MDTN on Systemic MK801-Evoked L-Glutamate Release (Study 3)

**Figure 3:**
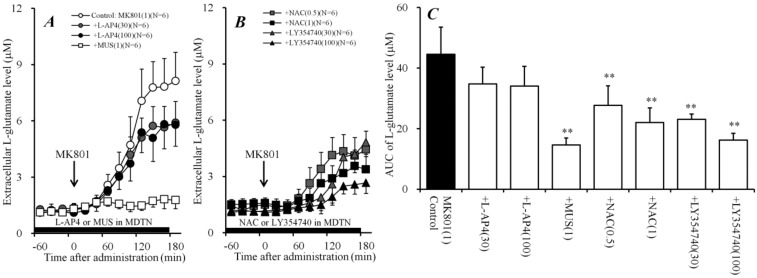
Effects of local administration of agonists of cystine/glutamate antiporter (Sxc), metabotropic glutamate receptors (mGluR), and GABA_A_ receptors into the MDTN on systemic MK801-evoked L-glutamate release in the mPFC. (**A**,**B**) indicate the effects of perfusion with L-AP4 (30 and 100 μM), muscimol (MUS) (1 μM) [5,6], NAC (0.5 and 1 mM), and LY354740 (30 and 100 μM) into the MDTN (black bars) on systemic MK801-evoked L-glutamate release in the mPFC, compared with MK801 alone (1 mg/kg, i.p.) from (**A**). Ordinates: mean ± SD (*n* = 6) of the extracellular L-glutamate level in the mPFC (μM), abscissa: time after administration of 1 mg/kg MK801 (min). Black arrows indicate the intraperitoneal injection of MK801. Microdialysis was conducted to measure the L-glutamate release in the mPFC. (**C**) indicates the AUC value of the extracellular L-glutamate level in the mPFC (μM) after MK801 injection from 0 to 180 min of (**A**,**B**). ** *P* < 0.01; relative to 1 mg/kg MK801 alone (control) using LMM with Tukey’s post hoc test.

To clarify the generator regions of the inhibitory effects of systemic administration of NAC on systemic MK801-evoked L-glutamate release, the effect of local administration of NAC into the MDTN on systemic MK801-evoked L-glutamate release was determined. Perfusion with NAC (0.5 and 1 mM) into the MDTN concentration-dependently reduced systemic MK801-evoked L-glutamate release [F_NAC_(2,15) = 16.2 (*P* < 0.01), and F_time_(9,135) = 189.0 (*P* < 0.01), F_NAC*time_ (18,135) = 26.2 (*P* < 0.01)] (Figure 3B,C).

Previous reports demonstrated that NAC enhanced the mGluRs [31,32,33], therefore, to study the effect of mGluRs in the MDTN on systemic MK801-evoked L-glutamate release, the concentration-dependent effects of local administration (perfusion) of II-mGluR agonist, LY354740 (30 and 100 μM) and III-mGluR agonist, L-AP4 (30 and 100 μM) into the MDTN on systemic MK801-evoked L-glutamate release were determined. Perfusion with LY354740 (30 and 100 μM) into the MDTN concentration-dependently reduced systemic MK801-evoked L-glutamate release [F_LY354740_(2,15) = 44.1 (*P* < 0.01), F_time_(9,135) = 133.0 (*P* < 0.01), and F_LY354740*time_ (18,135) = 24.3 (*P* < 0.01)] (Figure 3B,C). Contrary to II-mGluR, perfusion with L-AP4 (30 and 100 μM) into the MDTN did not affect systemic MK801-evoked L-glutamate release (Figure 3A,C).

It has been well established that the MDTN receives two types of GABAergic projections from the dorsal reticular thalamic nucleus (DRTN) and other MDTN regions [36], indeed; perfusion with GABA_A_ receptor agonist, muscimol (MUS: 1 μM) [5,6] into the MDTN reduced systemic MK801-evoked L-glutamate release [F_MUS_(1,10) = 62.2 (*P* < 0.01), F_time_(9,90) = 88.0 (*P* < 0.01), and F_MUS*time_(9,90) = 74.5 (*P* < 0.01)] (Figure 3A,C).

Therefore, these results suggest that thalamo-cortical glutamatergic transmission receives inhibitory regulation that is associated with Sxc, II-mGluR, and GABA_A_ receptors, but not III-mGluR in the MDTN. The selective regulation mechanism in the MDTN was studied in Study 5.

### 2.4. Effects of Perfusion of Activators of Sxc, mGluRs, and GABA_A_ Receptor into mPFC on Systemic MK801-Evoked L-Glutamate Release (Study 4)

**Figure 4:**
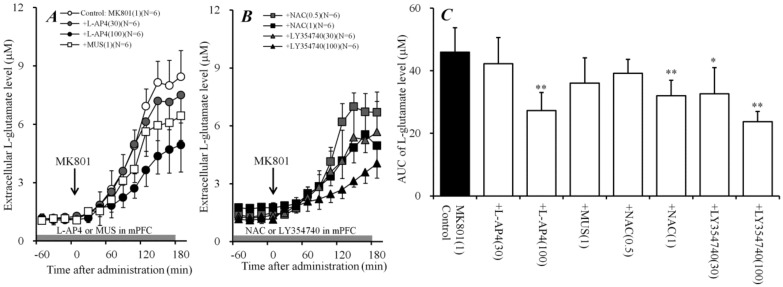
Effects of local administration of agonists of Sxc, mGluR, and GABA_A_ receptors into the mPFC on systemic MK801-evoked L-glutamate release in the mPFC. (**A**,**B**) indicate the effects of perfusion with L-AP4 (30 and 100 μM), MUS (1 μM) [5,6], NAC (0.5 and 1 mM), and LY354740 (30 and 100 μM) into the mPFC (gray bars) on systemic MK801-evoked L-glutamate release in the mPFC, compared with MK801 alone (1 mg/kg, i.p.) from (**A**). Ordinates: mean ± SD (*n* = 6) of the extracellular L-glutamate level in the mPFC (μM), abscissa: time after administration of 1 mg/kg MK801 (min). Black arrows indicate the intraperitoneal injection of MK801. Microdialysis was conducted to measure the L-glutamate release in the mPFC. (**C**) indicates the AUC value of the extracellular L-glutamate level in the mPFC (μM) after MK801 injection from 0 to 180 min of (**A**,**B**). * *p* < 0.05, ** p < 0.01; relative to 1 mg/kg MK801 alone (control) using LMM with Tukey’s post hoc test.

Study 3 identified the regulation mechanisms of the thalamo-cortical pathway in the MDTN. Similar to the MDTN, Sxc in the mPFC possibly inhibits thalamo-cortical glutamatergic transmission in the terminal region, since Sxc is widely expressed in central nervous system astrocytes [30]. Therefore, to clarify the generator regions of the inhibitory effects of systemic administration of NAC on systemic MK801-evoked L-glutamate release, the effect of local administration of NAC into the mPFC on systemic MK801-evoked L-glutamate release was determined. Similar to the MDTN, perfusion with NAC (0.5 and 1 mM) into the mPFC concentration-dependently reduced systemic MK801-evoked L-glutamate release [F_NAC_(2,15) = 7.4 (*P* < 0.01), F_time_(9,135) = 402.8 (*P* < 0.01), and F_NAC*time_(18,135) = 17.4 (*P* < 0.01)] (Figure 4B,C).

To determine the effects of mGluRs in the mPFC on systemic MK801-evoked L-glutamate release, the concentration-dependent effects of perfusion with L-(+)-2-amino-4-phosphonobutyric acid (L-AP4) (30 and 100 μM) and LY354740 (30 and 100 μM) into the mPFC on systemic MK801-evoked L-glutamate release were determined. Similar to the MDTN, perfusion with LY354740 (30 and 100 μM) into the mPFC concentration-dependently reduced systemic MK801-evoked L-glutamate release [F_LY354740_(2,15) = 15.4 (*P* < 0.01), F_time_(9,135) = 186.1 (*P* < 0.01), and F_LY354740*time_(18,135) = 21.5 (*P* < 0.01)] (Figure 4B,C). Contrary to the MDTN, perfusion with L-AP4 (30 and 100 μM) into the mPFC concentration-dependently reduced systemic MK801-evoked L-glutamate release [F_L-AP4_(2,15) = 10.4 (*P* < 0.01), F_time_(9,135) = 236.0 (*P* < 0.01), and F_L-AP4*time_(18,135) = 9.3 (*P* < 0.01)] (Figure 4A,C). Furthermore, thalamo-cortical glutamatergic transmission was not affected by the activation of GABAergic inhibition in the mPFC [5,6]; indeed, perfusion with 1 μM MUS into the mPFC [5,6] did not affect systemic MK801-evoked L-glutamate release (Figure 4A,C).

Therefore, the regulation mechanisms of thalamo-cortical glutamatergic transmission in the mPFC (terminal regions) are different from those in the MDTN. In particular, the thalamo-cortical glutamatergic pathway receives inhibitory regulation that is associated with Sxc, II-mGlu, and III-mGluR, but not GABA_A_ receptors in the mPFC. The selective regulation mechanism in the mPFC was studied in Study 6.

### 2.5. Effects of Local Administration of Modulators of Sxc, mGluRs, and GABA_A_ Receptor into MDTN on Local MK801-Evoked L-Glutamate Release in mPFC (Study 5)

The results in Studies 1–4 strongly suggest that systemic MK801-evoked L-glutamate release is generated by the inhibition of NMDAR in the MDTN; however, activations of Sxc by NAC in both the MDTN and mPFC possibly contribute to the prevention of hyper-glutamatergic transmission in the thalamo-cortical (from the MDTN to the mPFC) pathway induced by impaired NMDAR in the MDTN. In other words, the thalamo-cortical hyper-glutamatergic abnormality can modulate the Sxc-associated regulation systems. Therefore, to explore the regulation mechanisms of the thalamo-cortical pathway in MDTN, the effects of mGluRs and Sxc in the MDTN on L-glutamate release in the mPFC induced by the perfusion of 50 μM MK801 into the MDTN (local MK801-evoked L-glutamate release) were determined.

**Figure 5:**
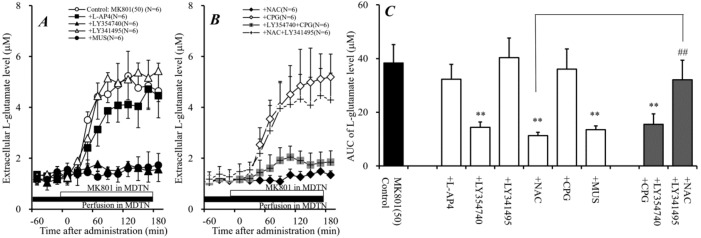
Effects of local administration of modulators of Sxc, mGluR, and GABA_A_ receptors into the MDTN on local MK801-evoked L-glutamate release in the mPFC. (**A**,**B**) indicate the effects of perfusion with L-AP4 (100 μM), LY354740 (100 μM), LY341495 (1 μM), NAC (1 mM), S)-4-carboxy-phenylglycine (CPG) (1 mM), and MUS (1 μM) into the MDTN on local MK801-evoked L-glutamate release in mPFC, compared with MK801 alone perfusion (control) from (**A**). Microdialysis was conducted to measure the L-glutamate release in the mPFC. In (**A**,**B**), ordinates: mean ± SD (*n* = 6) of the extracellular L-glutamate level in mPFC (μM), abscissa: time after administration of MK801 (min). Open bars: perfusion of 50 μM MK801 into the MDTN. Black bars: perfusion of 100 μM L-AP4, 100 μM LY354740, 1 μM LY341495, 1 mM NAC, 1 mM CPG or 1 μM MUS into the MDTN. (**C**) indicates mean ± SD (*n* = 6) of AUC value of the extracellular L-glutamate level in the mPFC (μM) during MK801 perfusion from 0 to 180 min of (**A**,**B**). ** *p* < 0.01; relative to control (perfusion of 50 μM MK801 alone), and ## *P* < 0.01; relative to perfusion of MK801 into the MDTN with NAC into the MDTN using LMM with Tukey’s post hoc test.

Local MK801-evoked L-glutamate release was inhibited by the perfusion of 100 μM LY354740 (II-mGluR agonist) [F_LY354740_(1,10) = 63.7 (*P* < 0.01), F_time_(9,90) = 59.1 (*P* < 0.01), F_LY354740*time_(9,90) = 43.3 (*P* < 0.01)], 1 mM NAC (cystine prodrug) [F_NAC_(1,10) = 85.9 (*P* < 0.01), F_time_(9,90) = 83.1 (*P* < 0.01), F_NAC*time_ (9,90) = 66.9 (*P* < 0.01)], and 1 μM MUS (GABA_A_ receptor agonist) [F_MUS_(1,10) = 71.2 (*P* < 0.01), F_time_(9,90) = 50.9 (*P* < 0.01), and F_MUS*time_(9,90) = 41.2 (*P* < 0.01)] into the MDTN, but was not affected by 100 μM L-AP4 (III-mGluR agonist), 1 μM LY341495 (II-mGluR antagonist) or 1 mM CPG (Sxc inhibitor) into the MDTN (Figure 5A–C).

The inhibitory effect of LY354740 on local MK801-evoked L-glutamate release in the mPFC was not inhibited by the perfusion of 1 mM CPG into the MDTN [F_LY354740_(3,20) = 31.1 (*P* < 0.01), F_time_(9,180) = 106.0 (*P* < 0.01), F_LY354740*time_(27,180) = 22.8 (*P* < 0.01)] (Figure 5B,C). The inhibitory effects of NAC on local MK801-evoked L-glutamate release was inhibited by the perfusion of 1 μM LY341495 [F_NAC_(3,20) = 26.4 (*P* < 0.01), F_time_(9,180) = 151.1 (*P* < 0.01), and F_NAC*time_(27,180) = 16.6 (*P* < 0.01)] (Figure 5B,C).

These results suggest that the inhibitory effect of NAC in the MDTN on Local MK801-evoked L-glutamate release is modulated by II-mGluR, whereas that of LY354740 (II-mGluR) is independent from Sxc.

### 2.6. Effects of Local Administration of Modulators of Sxc, mGluRs, and GABA_A_ Receptors into mPFC on Local MK801-Evoked L-Glutamate Release in mPFC (Study 6)

To explore the regulation mechanisms of the thalamo-cortical pathway in the mPFC, the effects of perfusion with modulators of mGluRs and Sxc into the mPFC on local MK801-evoked L-glutamate release were determined.

**Figure 6:**
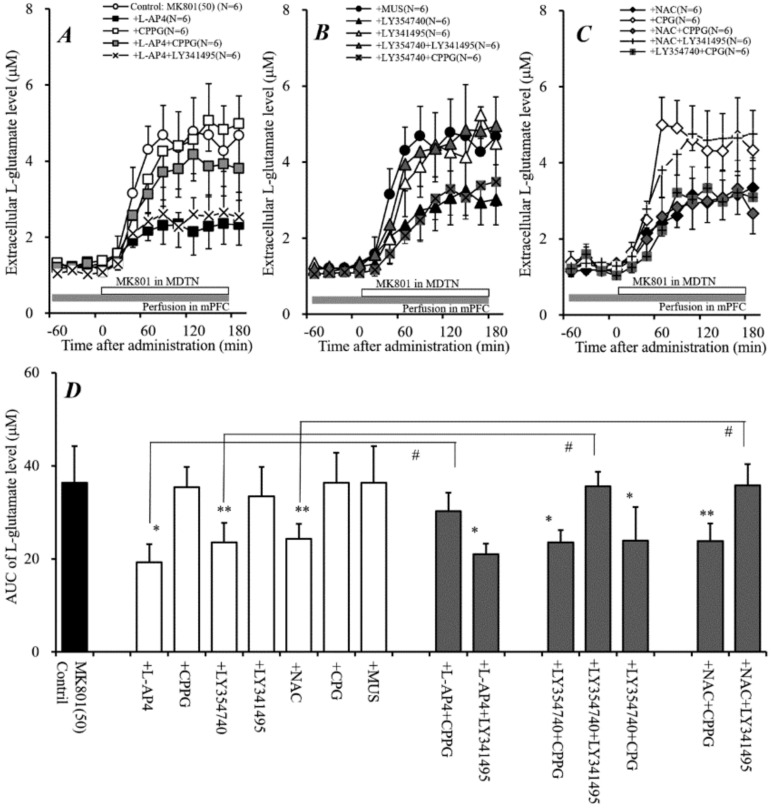
Effects of local administration of modulators of Sxc, mGluRs, and GABA_A_ receptors into the mPFC on local MK801-evoked L-glutamate release in the mPFC. (**A**–**C**) indicate the effects of perfusion with L-AP4 (100 μM), CPPG (100 μM), LY354740 (100 μM), LY341495 (1 μM), NAC (1 mM), CPG (1 mM), and MUS (1 μM) into the mPFC on local MK801-evoked L-glutamate release in the mPFC, compared with perfusion with MK801 alone from (**A**). Microdialysis was conducted to measure the L-glutamate release in the mPFC. In (**A**–**C**), ordinates: mean ± SD (*n* = 6) of the extracellular L-glutamate level in the mPFC (μM), abscissa: time after administration of MK801 (min). Open bars: perfusion of 50 μM MK801 into the MDTN. Gray bars: perfusion of 100 μM CPPG, 100 μM L-AP4, 100 μM LY354740, 1 μM LY341495, 1 mM NAC, 1 mM CPG, or 1 μM MUS into the mPFC. (**D**) indicates mean ± SD (*n* = 6) of AUC value of the extracellular L-glutamate level in the mPFC (μM) during MK801 perfusion from 0 to 180 min of (**A**–**C**). * *P* < 0.05, * *p* < 0.01; relative to Control (perfusion of 50 μM MK801 alone), and # *P* < 0.05; relative to perfusion of MK801 into the MDTN with L-AP4, LY354740, or NAC into the mPFC using LMM with Tukey’s post hoc test.

The inhibitory effects of perfusion with 100 μM L-AP4 (III-mGluR agonist) into the mPFC on local MK801-evoked L-glutamate release were inhibited by the perfusion of 100 μM (RS)-α-cyclopropyl-4-phosphonophenyl glycine (CPPG) (III-mGluR antagonist) [F_CPPG_(2,15) = 14.7 (*P* < 0.01), F_time_(9,135) = 85.3 (*P* < 0.01), and F_CPPG*time_(18,135) = 8.9 (*P* < 0.01)], but not affected by 1 μM LY341495 (II-mGluR antagonist) into the mPFC (Figure 6A,D). The inhibitory effect of perfusion of 100 μM LY354740 (II-mGluR agonist) into the mPFC on local MK801-evoked L-glutamate release was inhibited by the perfusion of 1 μM LY341495 [F_LY341495_(2,15) = 14.7 (*P* < 0.01), F_time_(9,135) = 85.3 (*P* < 0.01), F_LY341495*time_(18,135) = 8.9 (*P* < 0.01)], but not affected by 100 μM CPPG or 1 mM CPG (Sxc inhibitor) into the mPFC (Figure 6B,D). Therefore, II-mGluR and III-mGluR pre-synaptically inhibit thalamo-cortical glutamatergic transmission in the mPFC.

The inhibitory effects of perfusion with 1 mM NAC (cystine prodrug) into the mPFC on local MK801-evoked L-glutamate release were inhibited by the perfusion of 1 μM LY341495 [F_LY341495_(3,20) = 5.7 (*P* < 0.01), F_time_(9,180) = 200.0 (*P* < 0.01), and F_LY341495*time_(27,180) = 6.2 (*P* < 0.01)], but not affected by 100 μM CPPG into the mPFC (Figure 6C,D). Neither perfusion with 100 μM CPPG, 1 μM LY341495, 1 mM CPG, nor 1 μM MUS into the mPFC affected local MK801-evoked L-glutamate release (Figure 6A–D). These results suggest that the inhibitory effect of NAC in the mPFC on Local MK801-evoked L-glutamate release is modulated by both II-mGluR and III-mGluR.

### 2.7. Interaction between Local Administration of APZ and mGluR Antagonists into mPFC and MDTN on Systemic MK801-Evoked L-Glutamate Release in mPFC (Study 7)

Systemic administration of APZ improved schizophrenia-like behavior and inhibited L-glutamate release induced by NMDAR antagonists [34,35]; however, their detailed mechanisms remain to be clarified. Study 7 determined the effects of local administration of APZ into the mPFC and MDTN on systemic MK801-evoked L-glutamate release.

**Figure 7:**
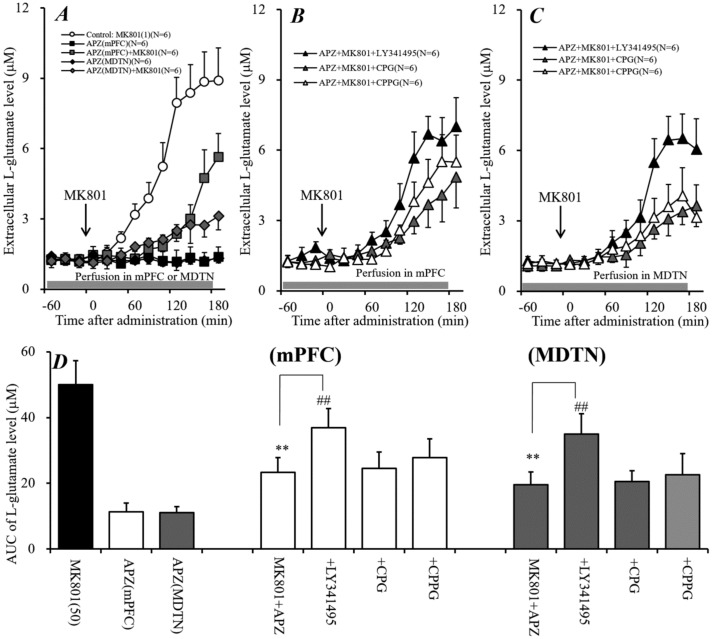
Interaction between local administration of Aripiprazole (APZ) and inhibitors Sxc and mGluR into the mPFC and MDTN on systemic MK801-evoked L-glutamate release in the mPFC. (**A**) indicates the effect of perfusion with APZ (20 μM) into the mPFC and MDTN (gray bar) on systemic MK801-evoked L-glutamate release in the mPFC. (**B**) indicates the effects of perfusion of LY341495 (1 μM), CPPG (100 μM), and CPG (1 mM) into the mPFC (gray bar) on the inhibitory effects of perfusion of 20 μM APZ into the mPFC on systemic MK801-evoked L-glutamate release in the mPFC, as compared with MK801 injection with APZ perfusion into the mPFC from (**A**). (**C**) indicates the effects of perfusion of LY341495 (1 μM), CPPG (100 μM), and CPG (1 mM) into the MDTN (gray bar) on the inhibitory effects of perfusion of 20 μM APZ into the MDTN on systemic MK801-evoked L-glutamate release in the mPFC, compared with MK801 injection with APZ perfusion into the MDTN from (**A**). In (**A**–**C**), ordinates: mean ± SD (*n* = 6) of the extracellular L-glutamate level in the mPFC (μM), abscissa: time after administration of MK801 (1 mg/kg, i.p.) (min). Black arrows indicate the intraperitoneal injection of MK801. (**D**) indicates the AUC value of the extracellular L-glutamate level in the mPFC (μM) after MK801 injection from 0 to 180 min of (**A**–**C**). ** *p* < 0.01; relative to Control (MK801 alone injection), ## *p* < 0.01: relative to MK801 injection with the perfusion of APZ into the mPFC or MDTN using LMM with Tukey’s post hoc test.

Perfusion of 20 μM APZ alone into the mPFC or the MDTN did not affect the extracellular L-glutamate level in the mPFC; however, perfusion of 20 μM APZ into the mPFC [F_APZ_(2,15) = 84.8 (*P* < 0.01), F_time_(9,135) = 207.5 (*P* < 0.01), and F_APZ*time_(18,135) = 82.1 (*P* < 0.01)] and the MDTN [F_APZ_(2,15) = 99.9 (P < 0.01), F_time_(9,135) = 227.6 (*P* < 0.01), F_APZ*time_(18,135) = 130.8 (*P* < 0.01)] inhibited systemic MK801-evoked L-glutamate release in the mPFC (Figure 7A,D).

To study the mechanisms of inhibition of APZ on systemic MK801-evoked L-glutamate release, the interaction between APZ and inhibitors of Sxc (CPG), II-mGluR (LY341495), and III-mGluR (CPPG) on systemic MK801-evoked L-glutamate release was determined. The inhibitory effects of perfusion of 20 μM APZ into the mPFC on systemic MK801-evoked L-glutamate release were inhibited by the perfusion of 1 μM LY341495 into the mPFC [F_LY341495_ (1,10) = 22.0 (*P* < 0.01), F_time_(9,90) = 166.8 (*P* < 0.01), and F_LY341495*time_(9,90) = 20.0 (*P* < 0.01)], but not affected by the perfusion with 1 mM CPG or 100 μM CPPG (Figure 7B,D). Similar to the mPFC, the inhibitory effects of perfusion of 20 μM APZ into the MDTN on systemic MK801-evoked L-glutamate release were inhibited by the perfusion of 1 μM LY341495 into the MDTN [F_LY341495_(1,10) = 25.7 (*P* < 0.01), F_time_(9,90) = 156.1 (*P* < 0.01), F_LY341495*time_(9,90) = 47.9 (*P* < 0.01)], but not affected by perfusion with 1 mM CPG or 100 μM CPPG (Figure 7C,D). These results suggest that the inhibitory effect of APZ in both the mPFC and MDTN on systemic MK801-evoked L-glutamate release is modulated by II-mGluR, but not by Sxc or III-mGluR.

## 3. Discussion

### 3.1. Mechanism of Systemic MK801-Evoked L-Glutamate Release in mPFC

Clinically, non-competitive NMDAR antagonists (e.g., phencyclidine, ketamine, and MK801) produce schizophrenic symptoms and exacerbate symptoms in schizophrenia patients [3,12,13,14]. The detailed pathophysiology of NMDAR antagonist-induced psychosis remains to be clarified. To clarify the pathophysiological mechanisms, many preclinical studies have demonstrated the abnormalities of glutamatergic transmission induced by non-competitive NMDAR antagonists. Systemic administration of non-competitive NMDAR antagonists phencyclidine [19], ketamine [21], and MK801 [22] increases L-glutamate release in the mPFC. The present study also demonstrated that the systemic administration of MK801 dose-dependently increased L-glutamate release in the mPFC (systemic MK801-evoked L-glutamate release). Contrary to systemic administration, local administration into the mPFC of MK801 [5,6,7,20,22] and ketamine [21] did not affect L-glutamate release in the mPFC. These contradictive demonstrations between systemic and mPFC local administrations of MK801 on L-glutamate release in the mPFC suggest that the mPFC is probably not a fundamental region for the systemic MK801-evoked L-glutamate release in the mPFC. 

**Figure 8:**
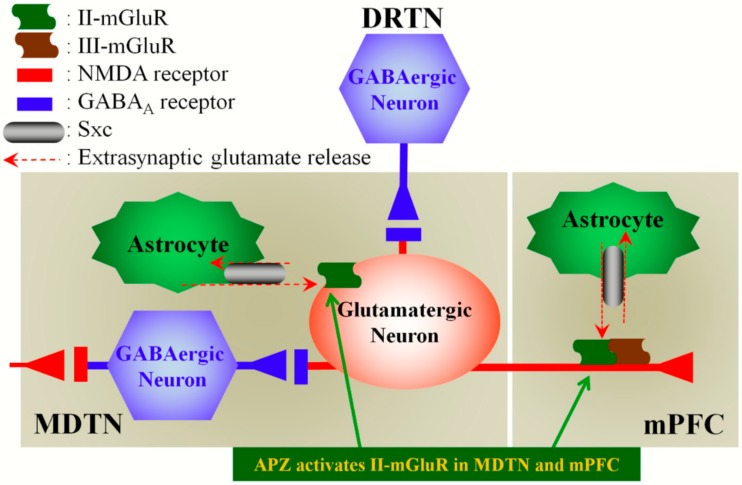
Our proposed hypothesis for the extended neural circuitry involved in MK801-induced L-glutamate release in the mPFC. MK801 inhibits tonically-active NMDAR (red squares) on MDTN GABAergic neurons (blue hexagon), which project to MDTN glutamatergic neurons (red circle). Inhibition of NMDAR in MDTN GABAergic neurons leads to disinhibition of MDTN glutamatergic neurons. This disinhibition results in the enhancement of MDTN glutamatergic signaling (increased L-glutamate release in the mPFC). II-mGluRs (green wave) in both the MDTN and mPFC are activated by glial-released L-glutamate through astroglial Sxc (gray ellipse). Activation of extra-synaptic II-mGluRs in the MDTN and mPFC results in the inhibition of MDTN glutamatergic projection. Activated III-mGluR (blown wave) in the mPFC pre-synaptically inhibits the activity of MDTN glutamatergic projection. APZ probably compensates hyper-activation of thalamo-cortical glutamatergic transmission via the activation of II-mGluR in both MDTN and mPFC.

To clarify the mechanisms of systemic MK801-evoked L-glutamate release, the present study determined the thalamo-cortical (MDTN–mPFC) glutamatergic transmission, since anatomical/functional studies suggest that the disturbance of the MDTN is particularly relevant for cognitive dysfunction [37]. Indeed, in this study, the perfusion of MK801 into the MDTN increased L-glutamate release in the mPFC (local MK801-evoked L-glutamate release), in a concentration- dependent manner. MDTN receives two types of GABAergic projections from the DRTN and other MDTN regions [36] (Figure 8). An activation of the GABA_A_ receptor in the MDTN (perfusion of MUS into the MDTN) inhibited both systemic and local MK801-evoked L-glutamate release in the mPFC, whereas the activation of the GABA_A_ receptor in the mPFC (perfusion of MUS into the mPFC) did not affect it. Thus, these results regarding the interaction between NMDAR and the GABA_A_ receptor in the MDTN suggest that NMDAR in the MDTN positively regulates GABA release in the MDTN. In other words, MDTN–mPFC glutamatergic transmission is probably activated by the disinhibition of GABAergic transmission in the MDTN via inhibition of NMDAR in the MDTN (Figure 8). To clarify our hypothesis regarding the interaction between glutamatergic and GABAergic transmissions in the MDTN, further studies are needed to examine the effects of MK801 on GABA release in the MDTN.

### 3.2. Regulation Mechanisms of MDTN–mPFC Glutamatergic Transmission

To clarify the regulation mechanisms of MDTN–mPFC glutamatergic transmission, the present study determined the effects of local perfusion with agonists and antagonists of II-mGluR, III-mGluR, and Sxc into the MDTN and mPFC on both systemic and local MK801-evoked L-glutamate releases in the mPFC.

The half maximal effective concentration (EC50) value of L-AP4 at III-mGluR is a nanomolar order, but L-AP4 possesses activity at other mGluR subtypes at concentrations below 1 mM [38]. The half maximal inhibitory concentration (IC50) value of CPPG at III-mGluR is a micromolar order, and CPPG shows approximately 30-fold selectivity for III-mGluR, as compared to other mGluRs [38]. In the present study, the activation of III-mGluR in the mPFC (perfusion with L-AP4 into the mPFC) inhibited both systemic and local MK801-evoked L-glutamate releases, whereas the activation of III-mGluR in the MDTN did not affect them. III-mGluR, which includes mGlu4R, mGlu7R, and mGlu8R, is localized in the presynaptic active zone, and negatively regulates the transmitter release [39]. Therefore, the MDTN–mPFC glutamatergic pathway receives inhibitory regulation in the mPFC of pre-synaptically expressed III-mGluR, but it is not regulated by III-mGluR in the MDTN.

II-mGluR, which includes mGlu2R and mGlu3R, is widely expressed in the central nervous system. In particular, mGlu3R is expressed in both pre- and postsynaptic or somato-dendritic regions, whereas mGlu2R is localized in the extra-synaptically axonal pre-terminal region [39]. In the present study, perfusion with LY354740 (II-mGluR agonist) into the mPFC and MDTN inhibited both systemic and local MK801-evoked L-glutamate releases. This study cannot deny the possibility that these inhibitions are probably induced by activations of II-mGluR and mGlu8R (III-mGluR), since the EC50 values of LY354740 at mGlu2R, mGlu3R, and mGlu8R are 5 nM, 24 nM, and 36 μM, respectively [38]. The inhibitory effects of perfusion with LY354740 into the MDTN on MK801-evoked L-glutamate release is considered to be mainly modulated by the activation of II-mGluR, but not by III-mGluR in the MDTN, since III-mGluR in the MDTN did not affect the MDTN–mPFC glutamatergic transmission. Contrary to the MDTN, based on the inhibitory effects of III-mGluR in the mPFC on MK801-evoked L-glutamate release, we should discuss in more detail the regulation of the MDTN–mPFC glutamatergic transmission in the mPFC that is associated with II-mGluR. To clarify the II-mGluR associated regulation, the present study determined the interaction among LY354740 (II-mGluR agonist), LY341495 (II-mGluR antagonist), and CPPG (III-mGluR antagonist) on local MK801-evoked L-glutamate release in the mPFC. The inhibitory effect of perfusion with LY354740 into the mPFC on MK801-evoked L-glutamate release was antagonized by perfusion with LY341495, but was not affected by CPPG into the mPFC. These results simply indicate that the inhibitory effects of the perfusion of LY354740 into the mPFC on MK801-evoked L-glutamate release is considered to be mainly modulated by its II-mGluR agonistic action in the mPFC. Therefore, taken together with histological demonstrations [39], the present study suggests that the MDTN–mPFC glutamatergic pathway is probably regulated by II-mGluR at both the post-synaptic region in the MDTN and the extra-synaptically pre-synaptic terminal region in the mPFC.

Recently, Copeland and colleagues demonstrated that the activation of II-mGluR by LY354740 inhibited GABAergic neuronal activities in the MDTN via astroglial transmission [40,41]. It has been established that LY354740 ameliorates psychotic behaviors in experimental models of schizophrenia [42]. Thus, both antipsychotics like LY354740 and psychometric MK801 probably disinhibit GABAergic transmission in MDTN–mPFC transmission. Taken together with these contradictive actions of LY354740 and MK801 in behavioral action and transmission, we hypothesized the enhancement of mPFC–MDTN inputs with the inhibition of MDTN–mPFC outputs play important roles in the pathophysiology regarding the cognitive dysfunction of schizophrenia. The details of our hypothesis should be clarified in our feature study.

Systemic and local (perfusion into the mPFC and MDTN) administrations of NAC (cystine prodrug) inhibited MK801-evoked L-glutamate release, whereas perfusion with CPG (Sxc inhibitor) into the mPFC and MDTN did not affect local MK801-evoked L-glutamate release in the mPFC. To clarify the broad inhibitory effects of Sxc on MDTN–mPFC glutamatergic transmission, the present study determined the interaction between Sxc and mGluR on local MK801-evoked L-glutamate release in the mPFC. In both the mPFC and MDTN, the inhibitory effects of NAC were reduced by the II-mGluR antagonist (LY341495), whereas the inhibitory effect of the II-mGluR agonist (LY354740) was not affected by the Sxc inhibitor (CPG). Furthermore, in the mPFC, the inhibitory effect of NAC was not affected by the III-mGluR antagonist (CPPG). These results suggest that the inhibitory effects of NAC on MK801-evoked L-glutamate release are inhibited by phasic activation of II-mGluR via released L-glutamate from astroglial Sxc [43]. Therefore, the MDTN–mPFC glutamatergic pathway receives extra-synaptic inhibitory regulation that is associated with II-mGluR, which is activated by the released L-glutamate from astroglial Sxc (Figure 8). In the mPFC, but not in the MDTN, the MDTN–mPFC glutamatergic pathway receives pre-synaptic inhibitory input that is associated with III-mGluR (Figure 8). Thus, MK801-evoked L-glutamate release in the mPFC is inhibited by the activation of the Sxc/II-mGluR complex in both the MDTN and mPFC, as well as III-mGluR independent from Sxc in the mPFC, but not in the MDTN (Figure 8).

### 3.3. Mechanisms of Action of APZ

APZ has a unique receptor binding profile for dopamine D2, serotonin 5-HT1A, and 5-HT2A receptors, without affecting mGluRs [8,9], and it especially acts as a partial agonist for dopamine D2 and serotonin 5-HT1A receptors [11,44]. These partial agonistic actions of APZ on dopamine D2 and serotonin 5-HT1A receptors result in the activation and inhibition of meso-cortical and meso-limbic dopaminergic transmission, respectively [7]. Several behavioral studies have demonstrated the effectiveness of APZ against the deficits of pre-pulse inhibition and social interaction induced by NMDAR antagonists [10,34,45]. The other II-mGluR agonist, LY379268, improved the positive and negative symptoms and cognitive disturbance of MK801-induced models [46]. The 5-HT1A receptor antagonist did not affect the effects of LY379268 on positive and negative symptoms [46]. Interestingly, the 5HT1A agonist and antagonist enhanced and inhibited the effects of LY379268 on cognitive disturbance [46]. Furthermore, the dopamine D2 receptor antagonist and 5-HT1A agonist inhibited NMDAR antagonist-induced L-glutamate release in the mPFC [47,48]. Taken together with these previous demonstrations, the partial 5-HT1A receptor agonistic action with the II-mGluR agonistic action of APZ probably contributes to the atypical antipsychotic action of APZ.

In this study, local administration of APZ into both the mPFC and MDTN reduced systemic MK801-evoked L-glutamate release in the mPFC. This inhibitory effect of APZ was abrogated by an II-mGluR antagonist (LY341495) but not by an III-mGluR antagonist (CPPG) or Sxc inhibitor (CPG). The activation of Sxc increases the production of glutathione, which removes reactive oxygen/nitrogen species and peroxides [29]; however, APZ had no or restricted scavenging effect [49]. Taken together with these previous demonstrations, the results in this study suggest that APZ inhibits MK801-evoked L-glutamate release, probably through the activation of II-mGluR (Figure 8). The direct effects of APZ on mGluR and Sxc remain to be clarified. The activation of II-mGluR stimulates the mitogen-activated protein kinase (MAPK) signaling pathway and MAPK/extracellular signal-related kinase kinase (MEK) inhibitors reduced II-mGluR induced long term depression [50,51]. Recent studies have demonstrated that APZ normalized the MAPK signaling pathway and activated the MAPK pathway, but it reduced the MK801-induced phosphorylation of MAPK [34,52]. These data highlight the Sxc/II-mGluR complex as a novel target, mediating the antipsychotic action of APZ. Further studies are needed to clarify the effects of APZ on mGluRs and MAPK pathways.

## 4. Materials and Methods

### 4.1. Chemical Agents

Aripiprazole (APZ), NMDAR antagonist MK801 [53], cysteine prodrug N-acetyl-L-cysteine (NAC) [54], and GABA_A_ receptor agonist muscimol (MUS) [53] were obtained from Wako Chemicals (Osaka, Japan). II-mGluR agonist LY354740 [55], antagonist LY341495 [55], III-mGluR agonist L-(+)-2-amino-4-phosphonobutyric acid (L-AP4) [55], antagonist (RS)-α-cyclopropyl-4-phosphonophenyl glycine (CPPG) [55], and Sxc inhibitor (S)-4-carboxy-phenylglycine (CPG) [56] were obtained from Tocris Bioscience (Bristol, UK).

All of the compounds were prepared on the day of the experiment. To study the effects of local administration of APZ, MK801, LY341495, LY354740, L-AP4, CPPG, CPG, NAC, and MUS, we perfused a modified Ringer’s solution (MRS) composed of (in mM) 145 Na^+^, 2.7 K^+^, 1.2 Ca^2+^, 1.0 Mg^2+^, and 154.4 Cl^−^, buffered to pH 7.4 with 2 mM phosphate buffer and 1.1 mM Tris buffer [20,57]. MK801, LY354740, L-AP4, CPPG, CPG, and MUS were dissolved in MRS directly. APZ and LY341495 were initially dissolved in 10 mM with dimethyl sulfoxide, and then diluted to 1 mM with MRS. The final concentrations of APZ, LY341495, and dimethyl sulfoxide were 20 µM, 1 µM, and 0.1% (vol/vol), respectively. To study the effects of systemic administration of MK801 (0.5 and 1 mg/kg) and NAC (50 and 100 mg/kg), MK801 (0.5 and 1 mg/mL), and NAC (50 and 100 mg/mL) dissolved in MRS were injected intraperitoneally (i.p.) in rats.

### 4.2. Preparation of Microdialysis System

All animal care and experimental procedures that were described in this report complied with the Ethical Guidelines established by the Institutional Animal Care and Use Committee at Mie University (No. 24-35). All studies involving animals are reported in accordance with the ARRIVE guidelines for reporting experiments involving animals [58]. A total of 228 rats were used in the experiments described here.

Male Sprague-Dawley rats (approximately 225~275 g, 7~8 weeks old, SLC, Shizuoka, Japan) were maintained in a controlled environment (22 ± 1 °C) on a 12-h dark/12-h light cycle. All rats were weighed prior to initiation of the study. Rats were anesthetized with 1.8% isoflurane and then placed in stereotaxic frame for 1 h. Concentric direct insertion type dialysis probes were implanted in the mPFC (A = +3.2 mm, L = +0.8 mm, V = −5.2 mm, relative to bregma) (0.22 mm diameter, 3 mm exposed membrane: Eicom, Kyoto, Japan) and MDTN (A = −3.0 mm, L = +0.9 mm, V = −6.2 mm, relative to bregma) (0.22 mm diameter, 2 mm exposed membrane: Eicom, Kyoto, Japan) [59]. Following surgery, rats were housed individually in cages during recovery and experiment, with food and water being provided ad libitum. Perfusion experiments commenced 18 h after recovery from isoflurane anesthesia [20,57]. The rat was placed into a system for freely moving animals (Eicom) equipped with a two-channel swivel (TCS2-23; Eicom). The perfusion rate was set at 2 μL/min in all experiments, using MRS [20,57]. Dialysate were collected every 20 min. Extracellular L-glutamate levels were measured at 8 h after starting the perfusion. The microdialysis experiments were carried out on awake and freely moving rats. To determine the effects of each agent, the perfusion medium was switched to MRS containing the target agent. Each dialysate was injected into the ultra-high-performance liquid-chromatography (UHPLC) apparatus.

After the microdialysis experiments, the brain was removed after cervical dislocation during overdose isoflurane anesthetizing. The location of the dialysis probes was verified by histological examination using 100 μm thick brain tissue slices (Vibratome 1000, Technical Products International INC, St. Louis, MO, USA).

### 4.3. Determination of Levels of L-Glutamate

L-glutamate levels were determined through UHPLC (xLC3185PU, Jasco, Tokyo, Japan) with fluorescence resonance energy transfer detection (xLC3120FP, Jasco) after dual derivatization with isobutyryl-L-cysteine and o-phthalaldehyde. Derivative reagent solutions were prepared by dissolving isobutyryl-L-cysteine (2 mg) and o-phthalaldehyde (1 mg) in 0.1 mL ethanol, followed by the addition of 0.9 mL sodium borate buffer (0.2 M, pH 9.0). An automated precolumn derivative was carried out by drawing up a 5 μL aliquot sample, standard or blank solution, and 5 μL of derivative reagent solution, and holding them in reaction vials for 5 min before injection. The derivatized samples (5 μL) were injected with an autosampler (xLC3059AS, Jasco). The analytical column (YMC Triat C18, particle 1.8 μm, 50 × 2.1 mm, YMC, Kyoto, Japan) was maintained at 45 °C and flow rate was set at 500 μL/min. A linear gradient elution program was performed over 10 min with mobile phase A (0.05 M citrate buffer, pH 5.0) and B (0.05 M citrate buffer containing 30% acetonitrile and 30% methanol, pH 3.5). The excitation/emission wavelengths of the fluorescence detector were set at 280/455 nm.

Where possible, we sought to randomize and blind the sample data. In particular, for the determination of the extracellular L-glutamate level, each sample was set on the autosampler according to a table of random numbers.

### 4.4. Data Analysis

All experiments were designed with equal sizes (*N* = 6) per groups. All values were expressed as mean ± SD. A *P* value less than 0.05 (*P* < 0.05) was considered to be statistically significant. The data were compared using the linear mixed effect model (LMM) using SPSS for Windows (ver 24, IBM, Armonk, NY, USA), followed by Tukey’s post hoc test using BellCurve for Excel (Social Survey Research Information Co., Ltd., Tokyo, Japan) when the F-value of the drug factor was significant. To represent the statistical significance of the drug factor as compared with LMM and Tukey’s post hoc test, the data (L-glutamate level) were expressed as the area under the curve (AUC_20–180 min_) values.

## 5. Conclusions

This study demonstrated that the inhibition of NMDAR in the MDTN contributes to increased L-glutamate release in the mPFC via activation of thalamo-cortical glutamatergic transmission induced by GABAergic disinhibition in the MDTN. Hyper-function of MDTN−mPFC glutamatergic transmission induced by reduced activation of NMDAR in the MDTN is normalized by the activation of Sxc, II-mGluR, and III-mGluR in the mPFC, as well as Sxc and II-mGluR in the MDTN. In the mPFC, the inhibitory effect of Sxc was dependent on both II-mGluR and III-mGluR, whereas the inhibitory effects of II-mGluR and III-mGluR were independent on Sxc activity. In the MDTN, the inhibitory effect of Sxc was also dependent on II-mGluR, whereas the inhibitory effect of II-mGluR was independent from Sxc activity. Therefore, released L-glutamate through Sxc activates these two mGluRs. APZ, an atypical antipsychotic agent, prevented NMDAR antagonist-induced L-glutamate release in the mPFC through the activation of the Sxc/II-mGluR complex in both brain regions. These results indicate that the regulation of MDTN−mPFC glutamatergic transmission by the Sxc/II-mGluR complex is a possible novel therapeutic target in antipsychotic medication against schizophrenia.

## Abbreviations

II-mGluR	group II metabotropic glutamate receptor
III-mGluR	group III metabotropic glutamate receptor
APZ	aripiprazole
CPG:	(S)-4-carboxyphenylglycine
CPP:	3-(2-carboxypiperazin-4-yl)-propyl-1-phosphonic acid
CPPG:	(RS)-α-cyclopropyl-4-phosphonophenyl glycine
DRTN:	dorsal reticular thalamic nucleus
FRET:	fluorescence resonance energy transfer
L-AP4:	L-(+)-2-amino-4-phosphonobutyric acid
LMM:	linear mixed model
MAPK:	mitogen-activated protein kinase
MDTN:	mediodorsal thalamic nucleus
MK801	5-methyl-10,11-dihydro-5H-dibenzo[a,d]cyclohepten-5,10-imine
NMDAR	glutamate/NMDA receptor
mPFC:	medial pre-frontal cortex
MUS:	muscimol
NAC:	N-acetyl-L-cysteine
Sxc:	system xc-
UHPLC:	ultra-high-performance liquid-chromatography
